# Delayed Presentation of Posttraumatic Diaphragmatic Hernia Masquerading as Recurrent Acute Asthmatic Attack

**DOI:** 10.1155/2017/5037619

**Published:** 2017-09-19

**Authors:** A. I. El-Yakub, U. M. Bello, A. A. Sheshe, H. U. Naaya

**Affiliations:** ^1^Department of Surgery, Aminu Kano Teaching Hospital, Kano, Nigeria; ^2^Department of Surgery, University of Maiduguri Teaching Hospital, Maiduguri, Nigeria

## Abstract

Diaphragmatic hernia following blunt abdominal injury is extremely rare and often diagnosed late. Missed diagnosis is also common with this condition. We herein present a delayed presentation of diaphragmatic hernia following blunt abdominal injury that was initially misdiagnosed as recurrent acute asthmatic attack due to repeated presentation with episodic difficulty in breathing.

## 1. Introduction 

Blunt traumatic rupture of the diaphragm is a serious injury that is often difficult to diagnose [1]. As many as 30% of diaphragmatic hernias present late [2]. The incidence is rising due to increasing surgeons experience and surgical subspecialization in our subregion [1]. Ambroise Paré in 1579 described the first case of diaphragmatic rupture in a French artillery captain who initially survived a gunshot wound of the abdomen but died 8 months later of a strangulated gangrenous colon, herniating through a small diaphragmatic defect that would admit only the tip of a small finger [3].

Diaphragmatic hernia following blunt abdominal injury is extremely rare and occurs in 0.8–1.6% of cases [2]. It is uncommon, yet associated with high morbidity or mortality when diagnosed late [4]. Previously, diaphragmatic hernia has presented in several ways leading to initial misdiagnosis of influenza [5] or pleural effusion [2] thereby resulting in delay in the actual diagnosis and with consequent poor outcome. No previous report however of diaphragmatic hernia presenting with features of recurrent acute asthmatic attack was made.

We herein present a case of delayed presentation of diaphragmatic hernia masquerading as recurrent asthmatic attack two years after sustaining blunt abdominal injury.

## 2. Case Presentation

A 40-year-old business man presented to general surgery outpatient clinic with one-and-half-year history of recurrent difficulty in breathing and chest tightness. He had no history of cough, orthopnea, paroxysmal nocturnal dyspnoea, body swellings, or fever and no gastrointestinal symptoms. On account of this, he was treated on several occasions in various hospitals as acute asthmatic attack with no relief of his symptoms. He had past history of road traffic accident with blunt abdominal injury and head injury 2 years prior to presentation that was managed conservatively. There was no other significant medical history.

On physical examination, he was found to be well-preserved young man. He had a reduced air entry on lower left hemithorax with audible bowel sounds on the same site of the chest.

**Figure 1:**
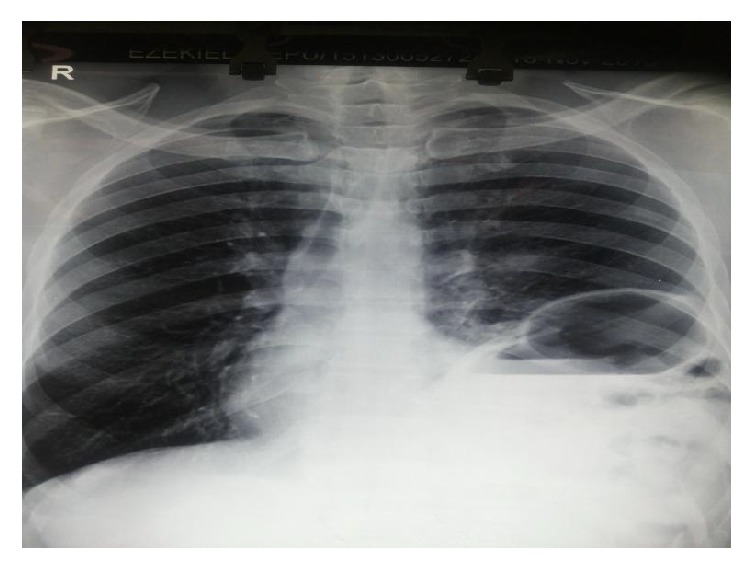
Chest X-ray showing air fluid levels and gastric fundal gas.

**Figure 2:**
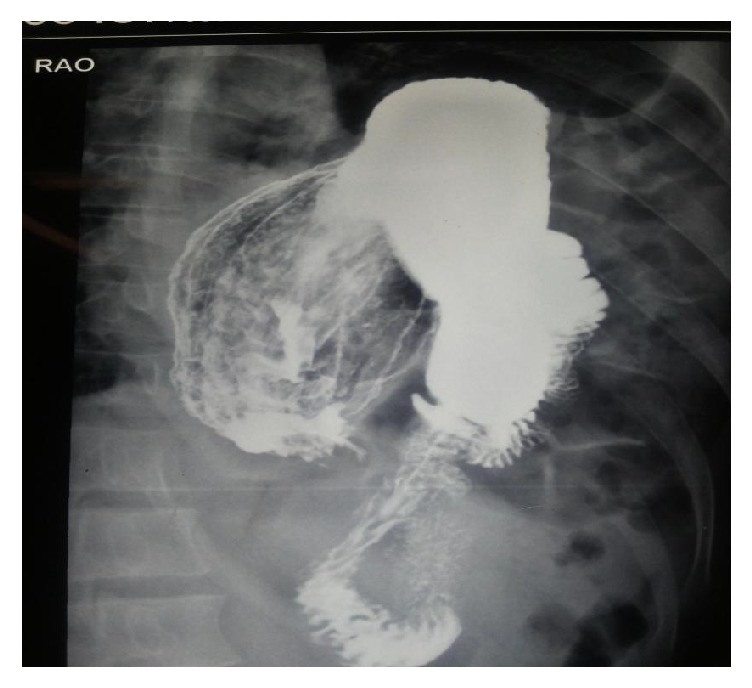
Barium enema showing transverse colon and splenic flexure in left hemithorax.

**Figure 3:**
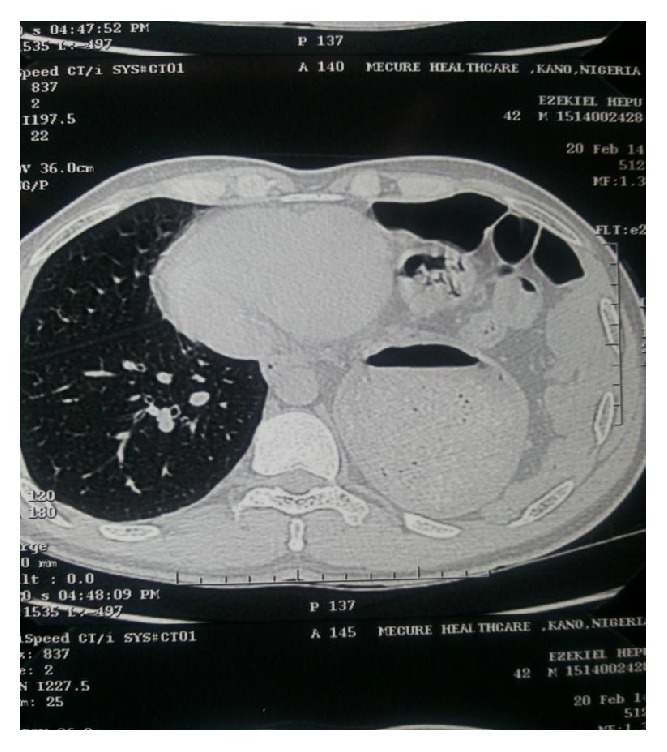
Computed tomography (CT) showing presence of stomach and bowel loops in the chest.

A chest X-ray shows obscured left hemidiaphragm, presence of gastric fundal gas, and air fluid level in left hemithorax (Figure 1). Barium enema shows transverse colon and splenic flexure in left hemithorax (Figure 2). Computed tomography (CT) shows presence of stomach and bowel loops in the chest (Figure 3).

**Figure 4:**
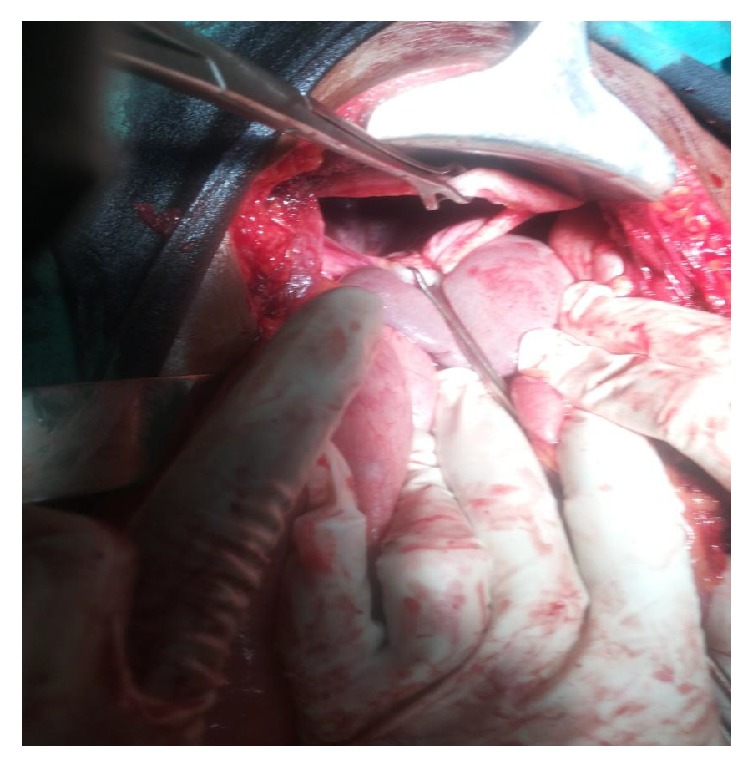
Operative finding of the diaphragmatic hernia.

He had laparotomy with intraoperative findings of 15 cm left diaphragmatic defect (Figure 4), with herniated stomach, transverse colon, omentum, and spleen; however, there were no adhesions with the lungs. The herniated organs were safely reduced into the abdomen and the hernial defect was repaired with nylon 1 suture in an interrupted manner. As there were no adhesions and bleeding in the chest, tube thoracostomy was not done. The patient did well and was discharged on postoperative day 8. He is still stable 3 years after surgery.

## 3. Discussion

Patients with posttraumatic diaphragmatic herniation frequently present months to years after the initial injury. The proposed reason for the delay in symptoms may be the presence of omentum and viscera plugging the diaphragmatic defect temporarily, allowing for symptomatic visceral herniation to occur months to years later [6]. Missed blunt diaphragmatic rupture results in herniation of the abdominal organs into the chest due to the abdominothoracic pressure gradient, which progressively enlarges the diaphragmatic defect [7]. Abdominal organs such as stomach, omentum, intestines, spleen, and liver are the commonest to herniate into the thoracic cavity [8]. Patients may therefore experience chest pain, recurrent shortness of breath, and gastrointestinal symptoms such as nausea and vomiting, epigastric discomfort, or abdominal pains. Bowel sounds may also be heard in left side of the chest. Our patient however had herniation of stomach, spleen, omentum, and transverse colon. Similarly, he presented with recurrent difficulty in breathing and chest tightness which made diagnosis of asthmatic attack to be erroneously made on several occasions in various hospitals. The episodic nature of the symptoms in our patient could be because of combination of large diaphragmatic defect and the lack of adhesions between the herniated organs and the lung. These two factors led to herniation of large segments of intra-abdominal viscera into the chest under condition of increase in intra-abdominal pressure such as straining or coughing leading to respiratory compromise which led to the patient presenting with episodic respiratory difficulty simulating asthmatic attack.

Previously, Edino and colleagues have reported a case of gastropleurocutaneous fistula in a patient with delayed diagnosis of left diaphragmatic hernia [9].

Chest X-ray is the initial investigation of choice in patients with suspected diaphragmatic hernia and has sensitivity of 30–62% [10]. Radiographic features suggestive of diaphragmatic hernia include an elevated hemidiaphragm, distortion or obscuring of the diaphragmatic margin, bowel shadows or air/fluid levels above the diaphragm, intrathoracic presence of the nasogastric tube, associated pleural collection, lung collapse, and contralateral mediastinal shift.

Other plain radiograph findings that may suggest possibility of diaphragmatic rupture include abnormalities such as multiple lower rib fractures, haemothorax, and pneumoperitoneum [11]. Occasionally, there may be initial misdiagnosis on chest X-ray necessitating further investigations [2]. Computed tomography is also helpful in detecting diaphragmatic hernia and has sensitivity of 14% to 82% and specificity of 87% [12].

Gastrointestinal contrast study may reveal evidence of bowel herniation into the thoracic cavity further increasing the diagnostic accuracy [13].

Our patient had herniation of the transverse colon on barium enema investigation. Others include upper gastrointestinal contrast studies, diagnostic peritoneal lavage, fluoroscopy, ultrasound, magnetic resonance imaging, laparoscopy, and intraperitoneal injection of radioisotopes [11].

Treatment of diaphragmatic hernia is surgical via laparotomy or thoracotomy. Acute cases are better managed via a laparotomy as this also rules out and treats associated intra-abdominal organ injuries. Delayed cases, however, are better treated via a thoracotomy or thoracoabdominal approach because of intrathoracic adhesions [2, 11]. Our patient was operated on via laparotomy; however no adhesion was encountered between the herniated organs and the lungs despite presenting after 2 years of the blunt abdominal injury. Repair of diaphragmatic defect is usually achieved with interrupted nonabsorbable sutures like nylon and Prolene in a single or double-layered fashion [11]. Mesh repair is used for large defect [11].

## 4. Conclusion

Despite the fact that diaphragmatic hernia following blunt abdominal injury is rare, it should be suspected in a patient presenting with recurrent difficulty in breathing with previous history of blunt abdominal injury.
